# Supplementary material for: A mechanistic account of serotonin’s impact on mood
Source: Nat Commun. 2020 May 11;11:2335. doi: 10.1038/s41467-020-16090-2 (PMC7214430; doi:10.1038/s41467-020-16090-2)
Supplement: Supplementary file 1 — Supplementary Information [file 41467_2020_16090_MOESM1_ESM.pdf]

A mechanistic account of serotonin's impact on mood

Michely et al.

Supplementary Material

	Placebo		SSRI		P_{value}
Gender	19 ♀ / 13 ♂		19 ♀ / 13 ♂		1.000
Age	24.9 ± 3.9		24.5 ± 4.1		0.710
BDI – II [day 1]	4.5 ± 5.4		3.7 ± 4.1		0.504
BDI – II [day 7]	4.7 ± 5.7		4.5 ± 4.6		0.828
BDI – II [day 7 – day 1]	0.2 ± 3.3		0.8 ± 3.5		0.537
SHAPS [day 1]	0.3 ± 0.9		0.3 ± 0.7		1.000
SHAPS [day 7]	0.6 ± 1.6		0.8 ± 2.5		0.770
SHAPS [day 7 – day 1]	0.3 ± 1.5		0.5 ± 2.1		0.738
STAI - state [day 1]	30.4 ± 8.6		30.0 ± 6.5		0.845
STAI - state [day 7]	33.1 ± 9.1		31.3 ± 6.7		0.358
STAI - state [day 7 – day 1]	2.8 ± 8.5		1.3 ± 5.7		0.423
STAI - trait [day 1]	33.2 ± 9.8		34.7 ± 6.7		0.487
STAI - trait [day 7]	34.7 ± 9.9		35.8 ± 7.3		0.598
STAI - trait [day 7 – day 1]	1.5 ± 5.0		1.2 ± 2.9		0.762
PANAS - positive [day 1]	31.3 ± 8.7		30.0 ± 8.0		0.532
PANAS - positive [day 7]	29.2 ± 10.5		28.2 ± 8.4		0.667
PANAS - positive [day 7 – day 1]	-2.0 ± 7.4		-1.7 ± 5.7		0.865
PANAS - negative [day 1]	11.5 ± 2.5		11.2 ± 1.5		0.588
PANAS - negative [day 7]	12.1 ± 3.4		11.1 ± 1.8		0.156
PANAS - negative [day 7 – day 1]	0.5 ± 3.2		-0.2 ± 1.8		0.298

Supplementary Table 1. *Affective state questionnaire data.*

Drug groups were matched for age and gender, and there was no baseline difference in any of the affective state questionnaires (assessed on day 1, pre-drug). Moreover, there was no drug effect on any of the affective state measures. BDI – II = Beck's Depression Inventory II (Beck et al., 1996), SHAPS = Snaith-Hamilton Pleasure Scale (Snaith et al., 1995), STAI = State-Trait Anxiety Inventory (Spielberger, 1983), PANAS = Positive and Negative Affective Scale (Watson et al., 1988).

learning maintenance test

day	session	block 1		block 2	test
1	I.a	1-4		5-8	
1	I.b	9-12<br>1-2 & 5-6		13-16<br>3-4 & 7-8	9-16
1	II	17-20<br>9-10 & 13-14	WoF	21-24<br>11-12 & 15-16	17-24
4	III	25-28<br>17-18 & 21-22		29-32<br>19-20 & 23-24	25-32
7	IV	33-36<br>25-26 & 29-30	WoF	37-40<br>27-28 & 31-32	33-40<br>25-32

Supplementary Figure 1. *Experimental design: session structure and image appearance.*

All subjects were exposed to an identical set of 40 images in the study, where image order (1-40) was randomly shuffled across participants.

Each session comprised two learning blocks (image numbers depicted in orange), where subjects encountered four novel images, two with a high (70%) and two with a low reward probability (30%). Feedback enabled subjects to learn about images' reward probabilities through trial-and-error. After the second learning block, subjects performed a test choice block (depicted in blue), where they chose between images from the two preceding learning blocks. Maintenance performance of learned reward associations was assessed during interleaved trials (depicted in olive) involving familiar images on subsequent sessions, where images learned about in session I re-appeared in session II, and images from session II in session III, etc. Notably, both learning and maintenance were assessed on choices between the same image pairs, with the time-lag between learning and maintenance test constituting the only difference. To prevent further learning, outcomes were not revealed in test block *and* maintenance trials, and subjects were instructed to rely solely on what they had learned previously about the images.

Additionally, sessions II and IV comprised a WoF draw between learning blocks, where each subject either won or lost a large monetary amount.

Session IV test block comprised 24 additional trials, on which subjects were asked to choose between images that were learned in the same block on the previous session, i.e., session III, but encountered as interleaved maintenance trials either before or after the WoF draw on the current session, i.e., session IV. This design feature allowed us to assess the impact of mood on reconsolidation of reward associations.

Session I included two additional learning blocks to familiarise subjects with the task. Thus, the session comprised two learning blocks during the first part (I.a), and two learning blocks during the second part (I.b), i.e., four in total.

Mood bias on learning was assessed on session II (images 17-20 vs. 21-24), and IV (33-36 vs. 37-40). Mood bias on reconsolidation was assessed on session IV (25-26 vs. 27-28, and 29-30 vs. 31-32).

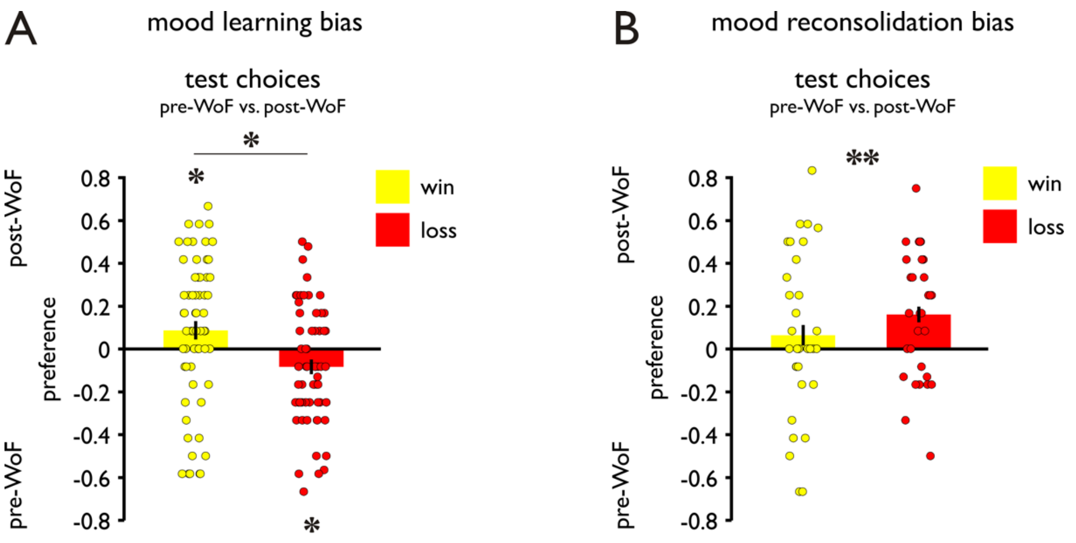

Supplementary Figure 2. *Effect of mood on learning and reconsolidation, across entire sample.*

(A) Proportion of test block choices between images encountered during preceding *learning* in different blocks, i.e., pre-WoF or post-WoF. This analysis compared choices between images with equal reward probabilities, i.e., high vs. high and low vs. low, and preference is computed as difference between percent choices of post-WoF images and pre-WoF images, as a function of WoF outcome.

Participants showed a preference for images encountered in better mood, favouring post-WoF images after a winning draw, and favouring pre-WoF images after a losing draw (win: p=0.047; loss: P=0.017; win vs. loss: p=0.004, n=62).

(B) Proportion of test block choices between images that had been encountered without feedback during preceding *reconsolidation* in different blocks, i.e., pre-WoF or post-WoF. This analysis involved choices between images with objectively similar reward probabilities, i.e., high vs. high and low vs. low, and preference is computed as difference between percent choices of post-WoF images and pre-WoF images, as a function of WoF outcome.

Contrasting a mood bias on learning, subjects preferred images that re-appeared post-WoF, irrespective of whether the WoF was won or lost (p=0.008, n=64). ** p<0.01, * p<0.05. Error bars indicate SEM.

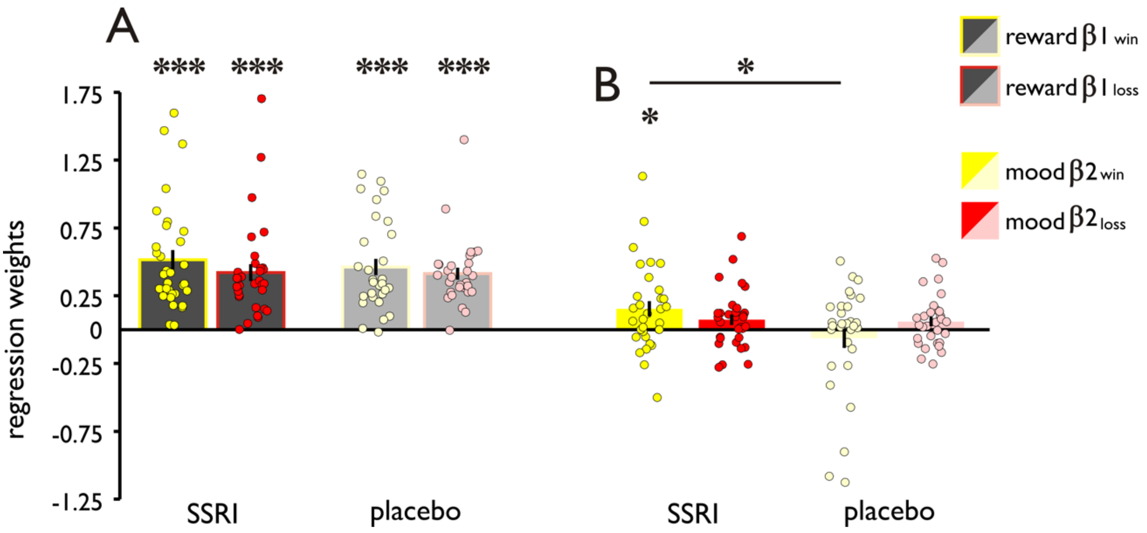

Supplementary Figure 3. *Logistic regression analysis (related to Fig. 3B, main paper).*

Logistic regression predicting choices on test block trials, accounting for impact of mood manipulation and number of rewards received for each image during learning with feedback. Predictors mood (depending upon the block in which the image was encountered during learning on WoF sessions) and difference of number of rewards received for the image pair with feedback during learning.

(A) Subjects were more likely to choose images for which they received more rewards during preceding learning (β_1; all $p<0.001$), but there was no difference between drug groups ($p=0.659$, $n=62$).

(B) Subjects preferred images encountered in a positive mood after a win ($\beta_{2_{positive}}$), and disfavoured images encountered in a negative mood after a loss ($\beta_{2_{negative}}$). There was a significantly enhanced positive, but not negative mood bias, in the SSRI as compared to the placebo group ($p=0.022$, $n=62$). *** $p<0.001$, ** $p<0.01$, * $p<0.05$, (*) $p<0.1$, n.s.=not significant (no difference across drug groups). Error bars indicate SEM.

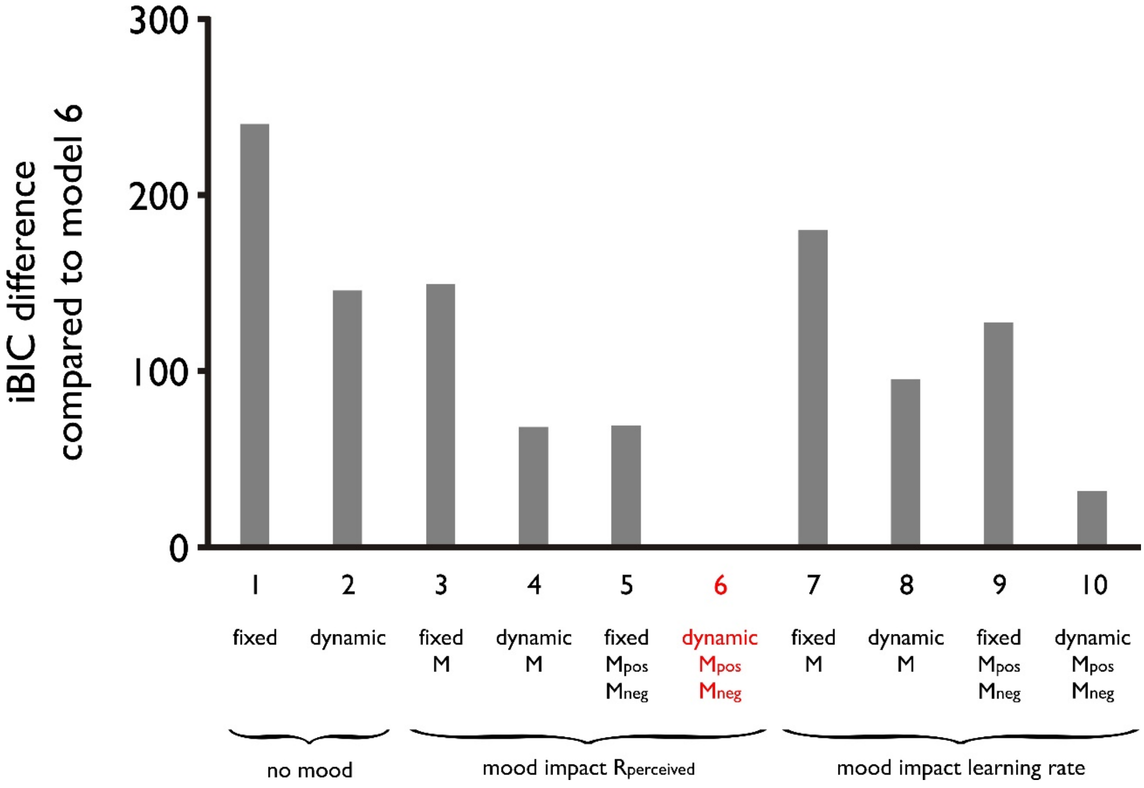

Supplementary Figure 4. *Model comparison.*

We compared 10 different models in terms of how well they explained subjects' choices in the task. For each model, iBIC scores (integrated Bayesian Information Criterion) are shown in comparison with the best-fitting model, i.e., model 6. A lower iBIC score indicates better fit with subjects' choices. Fixed = fixed learning rate; dynamic = dynamic learning rate; M = mood parameter; M_{pos} = positive mood parameter; M_{neg} = negative mood parameter. Note that models 1 and 2 did not include an impact of mood on learning, in models 3-6 mood impacts on subjective perception of reward during learning, and in models 7-10, mood impacts on rate of learning (cf. *Methods* for details).

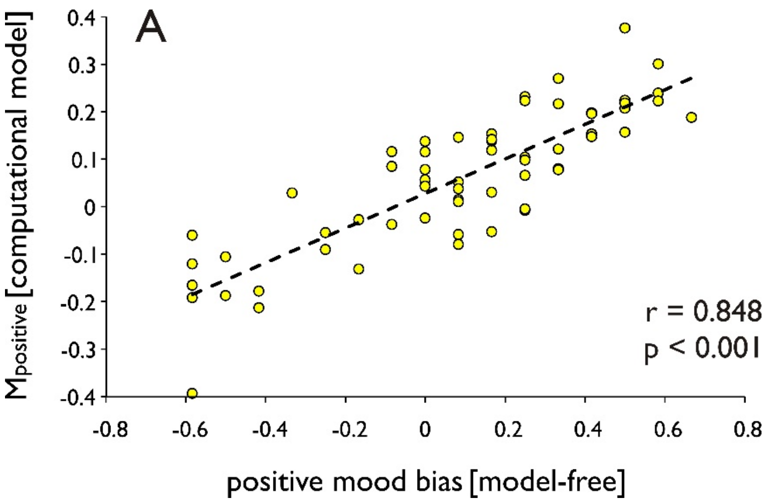

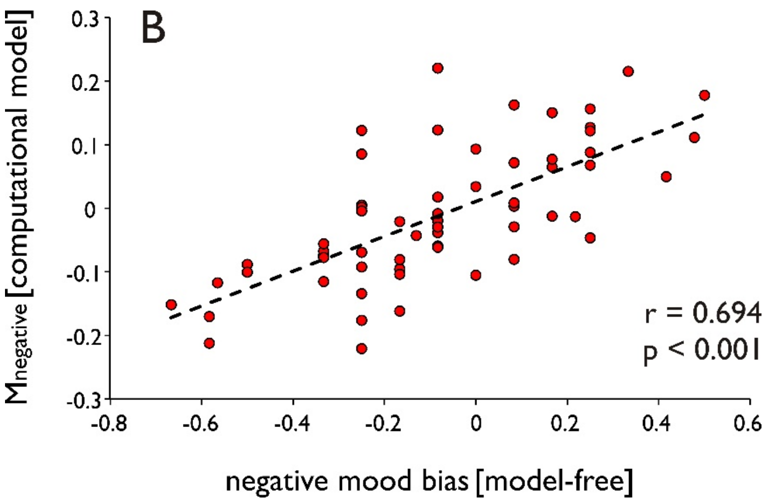

Supplementary Figure 5. *Relationship between model parameter estimates and model-free results.*

There was a strong positive correlation between model parameter estimates (derived from individually fitting the model parameters to each subject's choices; $M_{positive}$ & $M_{negative}$) and model-free mood bias results (derived from test block choices; cf. Fig. 3B), for both **(A)** positive (r=0.848, p<0.001) and **(B)** negative mood bias (r=0.694, p<0.001).

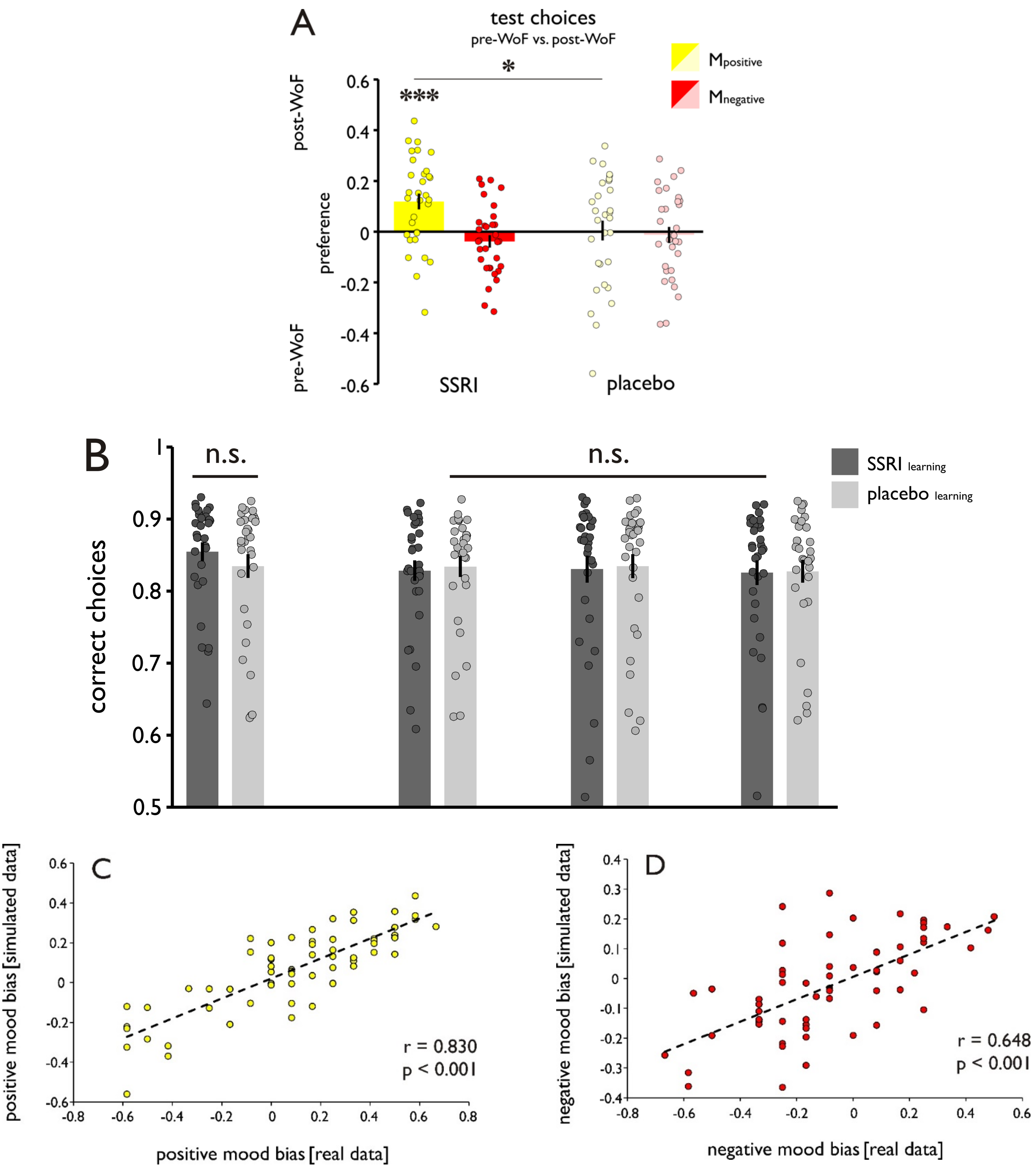

Supplementary Figure 6. *Results from simulated data, related to Fig. 2A/B & Fig. 3B, main paper.*

Generating simulated data based upon the model parameter estimates from the best-fitting model showed that the model captured core features of the real data. Note that for thess analyses, we simulated 100 data sets and averaged the results. The simulated data showed highly similar results for **(A)** mood bias (test block trials, high vs. high & low vs. low reward probability, cf. Fig. 3B, SSRI win: p=0.0006, SSRI vs. placebo win: p=0.026, n=62) and **(B)** learning performance (test block trials, high vs. low reward probability cf. Fig. 2A/B, SSRI vs. placebo, pre-drug: p=0.342, session II-IV: all p>0.783). Moreover, there was a strong positive correlation between mood bias results from real and simulated data, for both **(C)** positive (r=0.830, p<0.001) and **(D)** negative mood bias (r=0.648, p<0.001). *** p<0.001, ** p<0.01, * p<0.05, n.s.=not significant (no difference across drug groups). Error bars indicate SEM.

A

detected model (columns) / simulated model (rows)

	1	2	3	4	5	6	7	8	9	10
1	5									
2		5								
3			5							
4				5						
5					5					
6						5				
7							5			
8								5		
9									5	
10										5

B

**Model number**	**1**	**2**	**3**	**4**	**5**	**6**	**7**	**8**	**9**	**10**
Simulated data set 1	219	76	172	28	135	0	192	53	180	43
Simulated data set 2	235	89	164	32	133	0	208	61	184	39
Simulated data set 3	272	119	178	33	142	0	233	79	221	66
Simulated data set 4	299	114	214	29	169	0	239	62	225	59
Simulated data set 5	279	74	227	24	189	0	254	54	242	51

Supplementary Figure 7. *Validation of the model comparison procedure.*

We simulated 5 data sets using each model with its parameters fitted to subjects' real choices, and we applied the model comparison procedure to each data set.

(A) Each cell shows how many datasets generated by the model indicated on the vertical axis were detected as reflecting the model indicated on the horizontal axis. This analysis showed that the model comparison could always detect the model used for data simulation as the best-fitting model (5 times out of 5, indicated in red), confirming specificity of the model comparison procedure, i.e., model 6 is not recognized when it is not the true underlying model.

(B) This figure shows the analysis for data simulated using the winning model 6 in more detail. For each dataset simulated with model 6, model comparison detected model 6 as the best-fitting model, confirming sensitivity of the model comparison model procedure, i.e., model 6 is recognized when it is the true underlying model. For each model, iBIC scores are shown in comparison with the best-fitting model 6 (indicated in red).

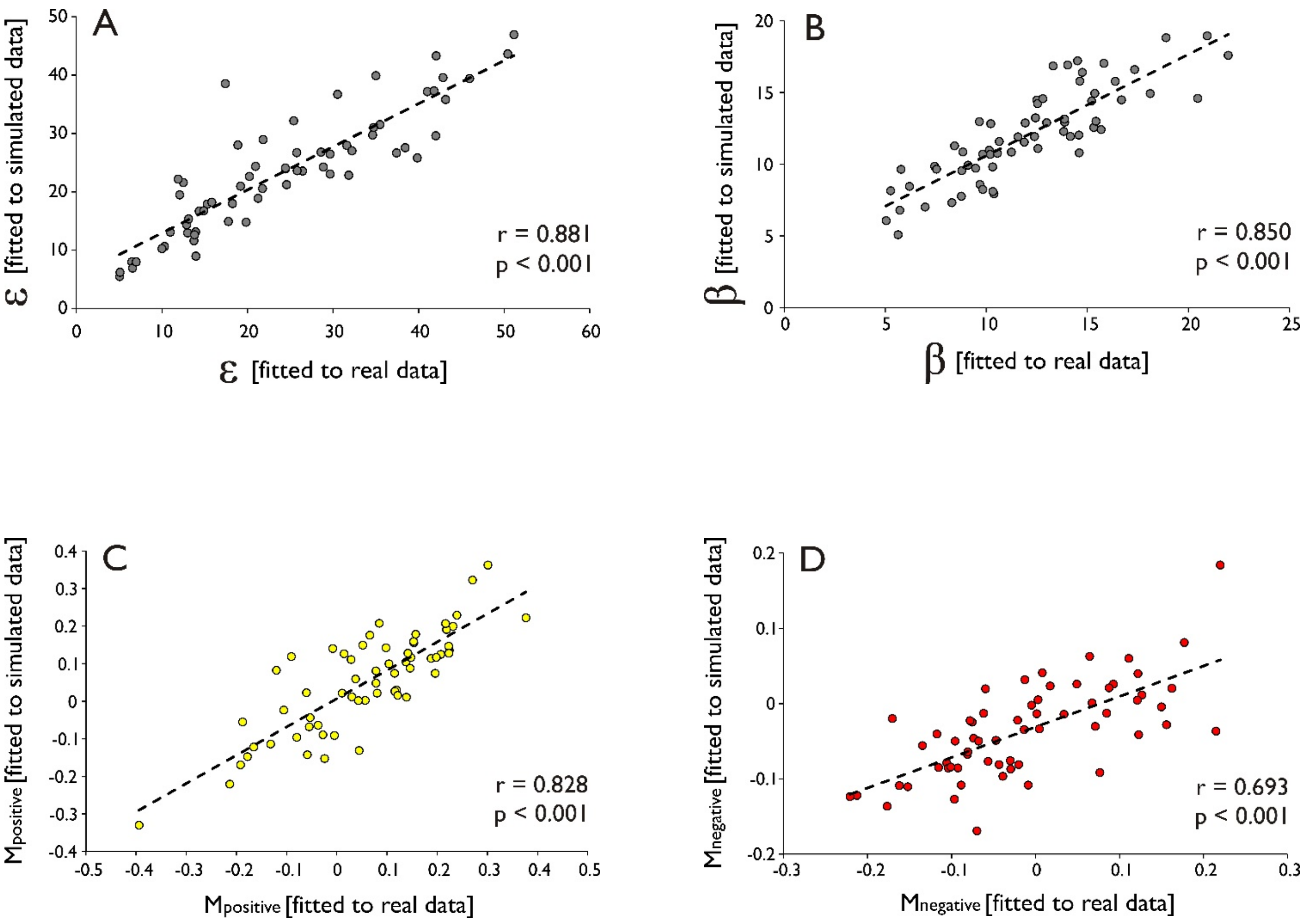

Supplementary Figure 8. *Recovery of model parameter estimates.*

Upon fitting the best-fitting model 6 to the simulated data, we found that model parameter estimates could be accurately recovered. This is indicated by a strong positive correlation between parameter estimates derived from fitting to real data (x-axis) and derived from fitting to simulated data (y-axis).

(A) ε = free parameter that determines initial learning rate, that dynamically changes as a function of the number of observed outcomes ($r=0.881$, $p<0.001$, cf. *Methods* for details), **(B)** β = inverse temperature parameter ($r=0.850$, $p<0.001$), **(C)** $M_{positive}$ = positive mood bias parameter ($r=0.828$, $p<0.001$), **(C)** $M_{negative}$ = negative mood bias parameter ($r=0.693$, $p<0.001$).

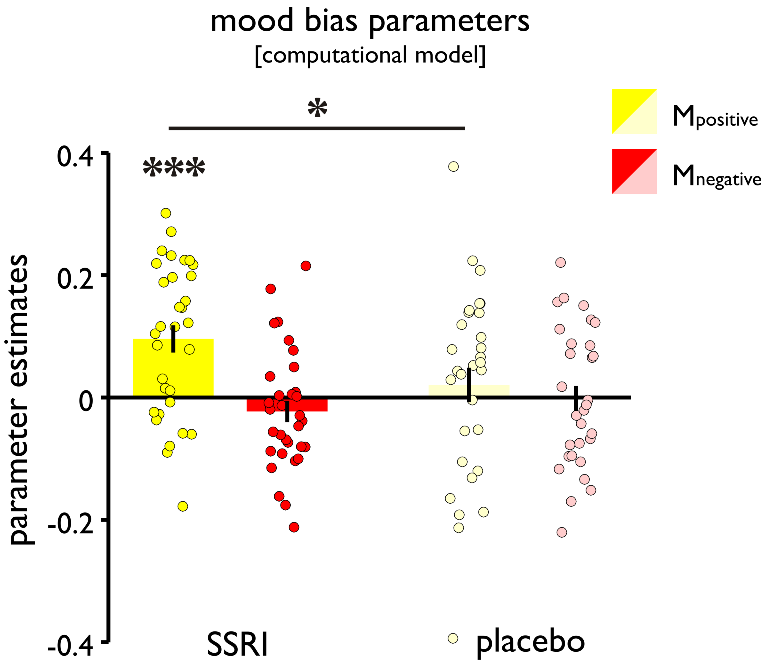

Supplementary Figure 9. *SSRIs boost the impact of positive mood on reward perception during learning.*

Individually fitting the model parameters to each subject's choices showed a significant drug difference in mood bias, driven by an elevated positive but not negative mood bias parameter in SSRI as compared to placebo treated subjects. This indicates that SSRIs boosted a subjective perception of reward in a positive mood (SSRI win: $p=0.0001$, SSRI vs. placebo win: $p=0.041$, $n=62$). *** $p<0.001$, ** $p<0.01$, * $p<0.05$. Error bars indicate SEM.

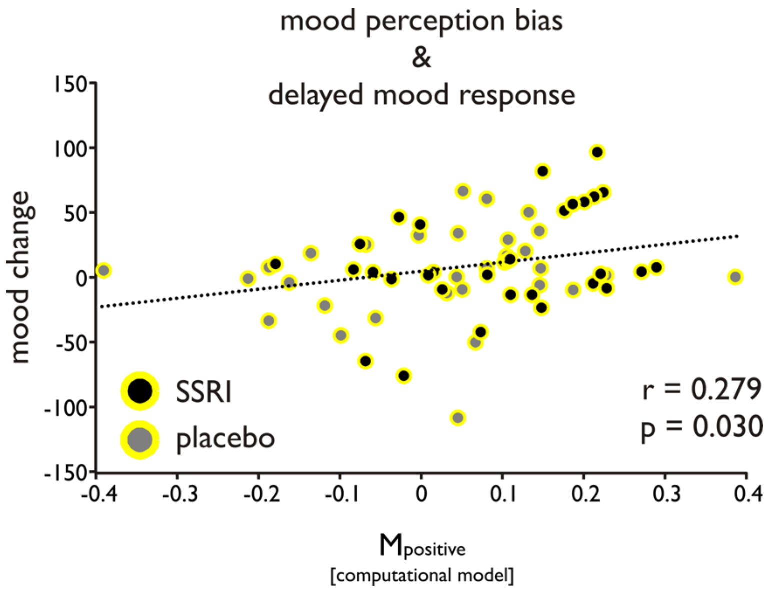

Supplementary Figure 10. *Positive mood bias and delayed positive mood response, related to Fig. 5E, main paper.*

Subjects with a greater positive mood bias parameter, derived from the computational model, showed a greater delayed positive mood response (block 1 to test block; $r=0.279$, $p=0.030$). Note that the positive mood bias parameter acts via boosting subjectively perceived reward during learning after winning the WoF, an effect significantly enhanced in the SSRI as compared to the placebo group.
